# Evaluation of the effect of botulinum toxin injection in aggravating or improving seborrheic dermatitis symptoms: A prospective, single‐arm clinical trial

**DOI:** 10.1111/srt.13478

**Published:** 2023-09-23

**Authors:** Afsaneh Sadeghzadeh Bazargan, Anahita Tabavar, Masoumeh Roohaninasab, Zahra Naeimaei Ali, Zeynab Tavana, Seyede Saba Mostafavi Montazeri, Alireza Jafarzadeh

**Affiliations:** ^1^ Department of Dermatology Rasool Akram Medical Complex Clinical Research Development Center (RCRDC) School of Medicine Iran University of Medical Sciences (IUMS) Tehran Iran; ^2^ Skin and Stem Cell Research Center, Tehran University of Medical Sciences Tehran Iran; ^3^ Student Research Committee, Iran University of Medical Sciences Tehran Iran; ^4^ Student Research Committee, Alborz University of Medical Sciences Karaj Iran

**Keywords:** botox, botulinum toxin, clinical trial, dandruff, seborrheic dermatitis, skin sebum

## Abstract

**Introduction:**

Considering the proven therapeutic effect of botulinum toxin and the pathophysiology of seborrheic dermatitis, conflicting hypotheses have been put forward regarding the effect of injection of this toxin on the improvement or exacerbation of seborrheic dermatitis. Because of the lack of consistent studies investigating this relationship, we decided to conduct this study to investigate the effect of local botulinum toxin injection on sebum production and improvement or worsening of seborrheic dermatitis lesions.

**Method:**

This study was a prospective, single‐arm clinical trial that involved the injection of botulinum toxin into 20 patients with complaints of skin wrinkles and simultaneous symptoms of seborrheic dermatitis. The trial was conducted at a dermatology clinic between March 2019 and March 2021. Two important characteristics of these patients were seborrheic dermatitis on the face or scalp and a referral for botulinum toxin injection to remove facial wrinkles. The Seborrheic Dermatitis Area and Severity Index (SDASI) was used to determine the severity of symptoms.

**Results:**

In study of 20 patients with an average age of 40 years, despite the decrease in the average scores of all examined criteria of seborrheic dermatitis symptoms in study, 1 month after botulinum toxin injection, no significant effect of using this toxin was seen on the improvement of patients’ symptoms (*p* value >0.05).

**Conclusion:**

Despite the emphasis of many studies on the effectiveness of botulinum toxin in reducing the activity of sebaceous glands, the use of botulinum toxin as a therapeutic modality for control the symptoms of seborrheic dermatitis is not suggested by this study. Conducting studies in which the location and technique of injection and the follow‐up intervals of patients in them are based on the standard of other studies, are the suggestions made by comparing the results and method of the current study with other studies.

**ABBREVIATIONS**
FDA: food and drug administration
SDASI: seborrheic dermatitis area and severity index

## 1 INTRODUCTION

Seborrheic dermatitis is a common skin disorder related to the growth of fungus in the skin, which affects about 4% of the world's population. This disease is more common in men than women, which can happen both in infancy and at any age after puberty.^1^ In dermatology, seborrheic dermatitis is defined as a chronic relapsing inflammatory skin disorder clinically characterized by scaling and erythematous patches with ill‐defined borders.^2^

Seborrheic dermatitis is a clinical diagnosis based on the location and appearance of the lesions,^3^ which in most cases involves the scalp, the central part of the face and the upper part of the chest, which is rich in sebaceous glands, and in adolescents and adults, often it appears as scaling of the scalp (dandruff). Seborrheic dermatitis may also cause mild erythema of the nasolabial fold, which is often accompanied by scaling.^1^, ^4^

However, in relation to botulinum toxin, it should be known that the injection of this compound for the treatment of facial wrinkles, is the most common cosmetic procedure performed in the United States.^5^ Botulinum toxin is one of the natural compounds that was first noticed due to its toxic properties, but today it is used as medicine.^6^

Regarding the effect of botulinum toxin injection on the symptoms of seborrheic dermatitis, there are two conflicting theories in the articles. In the first theory, the proven effect of botulinum toxin in the dermis layer on reducing the amount of production and secretion of skin sebum, regulating the secretion of skin neurotransmitters and blood flow under the skin through mediators.^7^ But in the second theory, following the intramuscular injection of botulinum toxin, by reducing muscle movement and facial expression, the discharge of sebum in the skin is delayed and causes the symptoms of seborrheic dermatitis to worsen.^6^

Therefore, considering the proven therapeutic effects of botulinum toxin and the pathophysiology of seborrheic dermatitis, the contradictory hypotheses raised regarding the effect of injection of this toxin on the improvement or exacerbation of seborrheic dermatitis lesions and the lack of coherent studies in the field of exploring this relationship, we decided to conducting this study, for evaluate the effect of local botulinum toxin injection on sebum production and improvement or exacerbation of seborrheic dermatitis lesions.

## 2 MATERIALS AND METHODS

### 2.1 Patients

This research was conducted in the form of a prospective, single‐arm clinical trial. At the beginning of the study, necessary permits were obtained to collect information from the patients. The research sample in this study consists of all patients referred to the dermatology clinic, from March 2019 to March 2021, who received botulinum toxin injection for removal of facial wrinkles and have Scalp or facial seborrheic dermatitis at the same time. The method of injection involved the administration of 150 units of Clostridium botulinum toxin type A (MASPORT®) into specific facial muscles, including the frontalis (forehead), procerus (between the eyebrows), orbicularis oculi (around the eyes) and corrugator muscles (between the eyebrows). Sampling of the study was done by convenience method and by examining all the available samples regarding to inclusion and exclusion criteria.

The exclusion criteria:

- The patients undergoing other treatment to control the symptoms of seborrheic dermatitis.
- The patients have lost to follow up after one month of injection through a repeat visit.

During the conduct of this study, after obtaining verbal permission and explaining the objectives of the study to the patients, information such as demographic data (age and gender), history of diabetes, neurological diseases, thyroid diseases, drug use and history and frequency of Botox injections, was recorded.

### 2.2 Assessment method

The severity of seborrheic dermatitis symptoms including skin erythema, scaling and skin sebum in the scalp and face was obtained using a Likert scale of 0–3 (zero means no lesion and 3 means the most severe condition of the symptoms) and also the extent of the lesions was recorded by a Likert scale of 1–5 (1: less than 10%, 2: 11%–30%, 3: 31%–50%, 4: 51%–70% and 5: more than 70%) based on the Seborrheic Dermatitis scoring criteria Area and Severity Index (SDASI).^8^

This action was done at the beginning of the visit and one month after one injection, based on the physician's diagnosis. The final severity of seborrheic dermatitis in each area was obtained by multiplying the sum of the scores of skin erythema, skin sebum and scaling in the extent of the lesions of that area. This procedure also was done for measure improvement or aggravation of seborrheic dermatitis in patients.

### 2.3 Data analysis

After collecting the mentioned information, all the data were entered into SPSS software and statistically analysed to measure the effect of botulinum injection on seborrheic dermatitis lesions. The demographic information required in the study population was extracted by taking the history of the patients at the time of visit and recorded in the checklists for each of these patients based on the variables.

Qualitative data were reported using the percentage and frequency index, and quantitative data were reported as Mean + SD. K‐S test was used to measure the normality of data distribution. In case of normal data distribution, Paired *T* test was used and in case of non‐normal data distribution, Wilcoxon test was used to evaluate the effect of botulinum toxin injection on the severity and extent of seborrheic dermatitis symptoms.

Statistical analysis of data and graphs were done using SPSS version 26 software, and the significance level was considered with *p*‐value less than 0.05.

### 2.4 Ethical principles

During the implementation of this study, the information related to all patients was kept with the researchers. This study registered in the Ethics Committee and code of the study is ‘IR.IUMS.FMD.REC.1401, 372’. The researchers were committed and adhered to the principles of the Helsinki Convention and the Ethics Committee of the Iran University of Medical Sciences in all stages.

## 3 RESULTS

**FIGURE 1:**
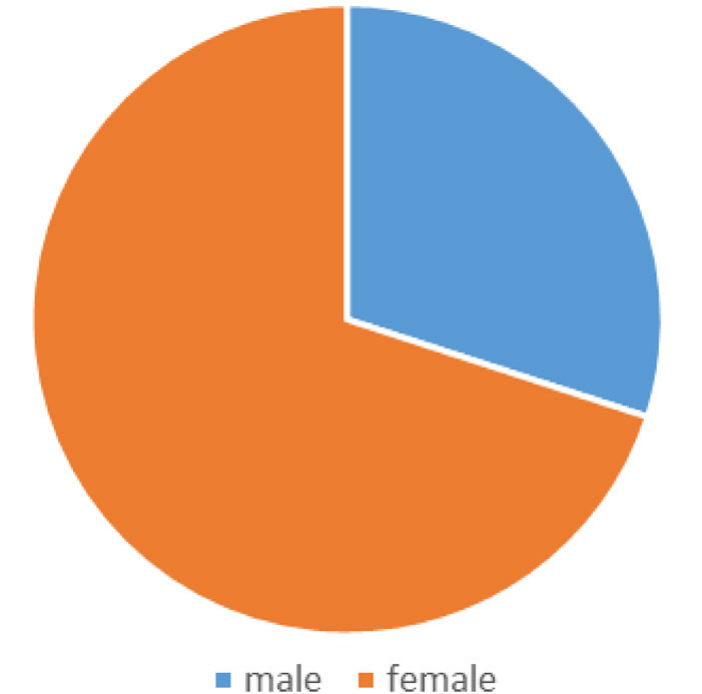
Gender distribution chart of patients.

At the beginning of the research, 34 patients were included in the study based on the determined inclusion criteria, but due to the patient loss to follow up, one month after the injection for a return visit, as one of the exclusion criteria, the information related to 20 patients were evaluated, in the end. In terms of gender, 6 patients (30%) were male and 14 patients (70%) were female. The gender distribution of patients can be seen in Figure 1.

**CAPSULE SUMMARY**

**What is known?**

Seborrheic dermatitis is a common skin condition characterised by itching, redness and scaling on the head, face and chest areas. To date, numerous systemic and local treatments have been proposed for this condition, with varying degrees of improvement and relapse. On the other hand, the injection of botulinum toxin has many applications in dermatology, for example, to improve wrinkles, to control excessive sweating and so on. As for the role of botulinum toxin injection in controlling the symptoms of seborrheic dermatitis, the results of previous studies are contradictory.

**What does this study add?**

This prospective, single‐arm clinical trial examined patients who presented symptoms of seborrheic dermatitis when referred for botulinum toxin injection to control skin wrinkles. The study aimed to assess the improvement in seborrheic dermatitis symptoms after the injection of botulinum toxin, and the results showed a decrease in severity indicators during the 1‐month follow‐up period. However, this difference was not statistically significant

The average age of the patients was 40.05 (±11.56) years, of which the youngest patient was 26 years old and the oldest patient was 67 years old. Patients were divided into 4 age groups and the highest frequency was in the age group of 26–37 years with 55%. The frequency and percentage distribution of patients in different age ranges can be seen in Table 1.

**TABLE 1:** Distribution table of the frequency of patients in different age ranges.

Age ranges	Frequency	Percentage
26–37	11	55
38–47	3	15
48–57	5	25
58–67	1	5
Total	20	100

**FIGURE 2:**
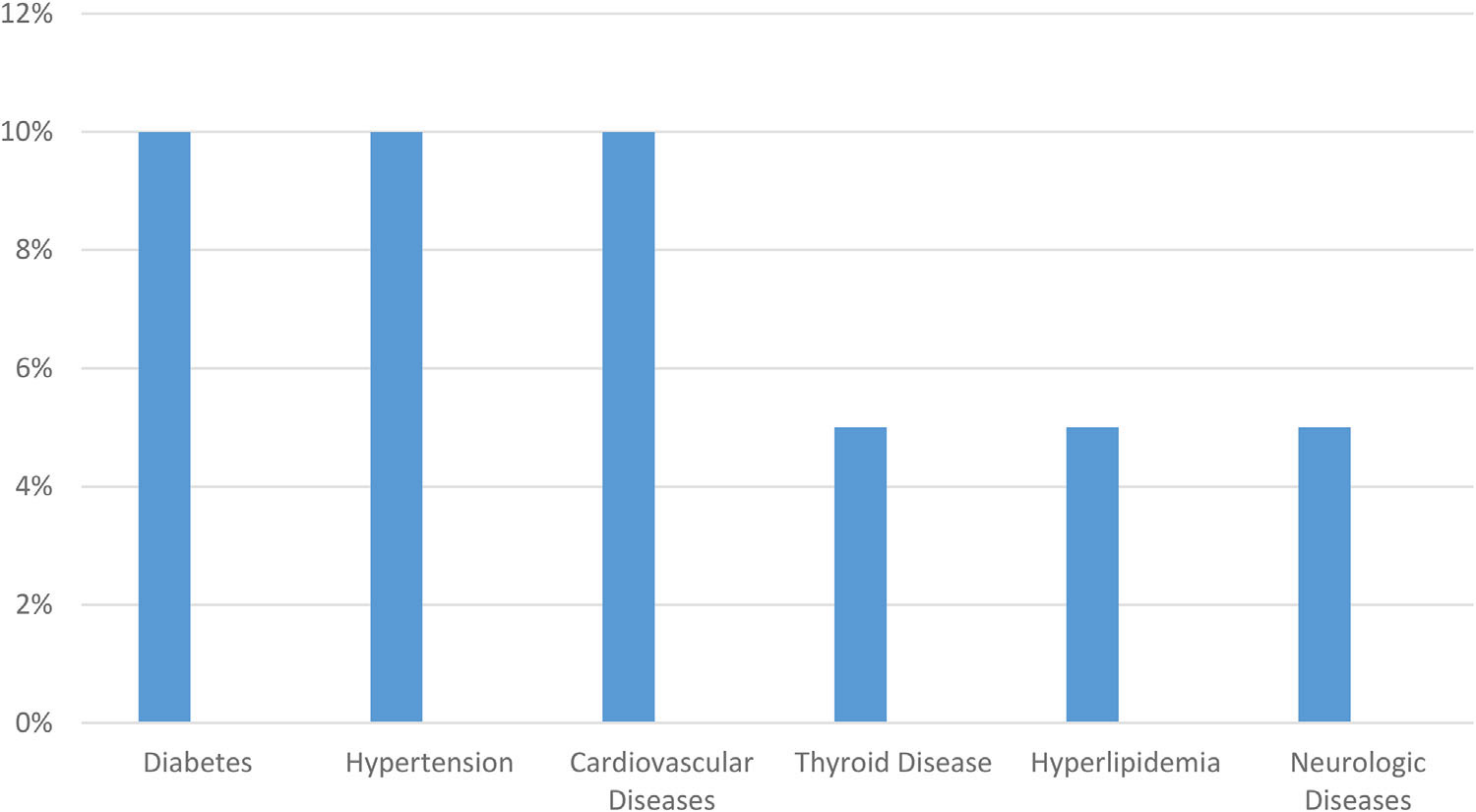
Distribution chart of the frequency of underlying diseases among the patients.

The most frequent underlying diseases were diabetes, hypertension and cardiovascular diseases each with two people (10%) of the patients, followed by thyroid diseases, hyperlipidaemia and neurological diseases with 1 person each (5%) of the patients. The only neurological disease detected in the patients' records was multiple sclerosis (MS). Figure 2 shows the frequency distribution of underlying diseases among patients.

The drugs used by the investigated patients included metformin, insulin, levothyroxine, losartan and atorvastatin, each with a frequency of 1 person (5%).There was a history of receiving botulinum toxin in 11 patients (55%) and the average number of times of receiving botulinum toxin among patients was calculated to be 1.4 ± 1.67 times.

Tables 2 and 3 show the average scores obtained from the examined criteria among patients at the first visit and 1 month after Botox injection in the scalp and facial skin, respectively.

**TABLE 2:** The table of average scores obtained from the studied criteria based on SDASI between patients at the first visit and 1 month after botulinum toxin injection in the scalp.

Measurement criteria		At the first visit	One month after Botox injection
Scalp	Skin erythema	0.25 (±0.44)	0.15 (±0.37)
	Skin sebum	1.35 (±1.04)	1.25 (±1.16)
	Scaling	1.60 (±0.68)	1.40 (±0.68)
	Extent of lesions	2.10 (±1.02)	1.15 (±1.23)
	Overall severity of seborrheic dermatitis	7.70 (±7.15)	6.60 (±6.76)

**TABLE 3:** The table of average scores obtained from the studied criteria based on SDASI between patients at the first visit and 1 month after botulinum toxin injection in the facial skin.

Measurement criteria		At the first visit	One month after Botox injection
Facial skin	Erythema	0.25 (±0.44)	0.20 (±0.41)
	Skin sebum	0.85 (±0.81)	0.80 (±0.83)
	Scaling	0.95 (±0.83)	0.80 (±0.77)
	Extent of lesions	1.65 (±0.88)	1.55 (±0.95)
	Overall severity of seborrheic dermatitis	4.05 (±4.55)	3.45 (±4.55)

The average of all criteria was associated with a decrease in the Likert scale number in the assessment one month after injection, but this decrease was not a statistically significant change in any of the criteria (*p*‐value >0.05).

In the evaluation of the scalp, despite the decrease in the average scores, in all measures of severity and extent of lesions; in none of the measures of skin erythema (*p* value = 0.157), skin sebum (*p* value = 0.317), scaling (*p* value = 0.248) and extent of lesions (*p* value = 0.739) did not show a statistically significant difference.

The overall severity of seborrheic dermatitis in the scalp 1 month after Botox injection compared to the first visit, despite the average decrease from 7.7 (±7.15) to 6.6 (±6.76), there was no statistically significant difference (*p* value = 0.528).

The average of all criteria was associated with a decrease in the Likert scale number in the assessment 1 month after injection, but this decrease was not a statistically significant change in any of the criteria (*p*‐value >0.05).

In the evaluation of the scalp, despite the decrease in the average scores, in all measures of severity and extent of lesions; in none of the measures of skin erythema (*p* value = 0.157), skin sebum (*p* value = 0.317), scaling (*p* value = 0.248) and extent of lesions (*p* value = 0.739) did not show a statistically significant difference.

**FIGURE 3:**
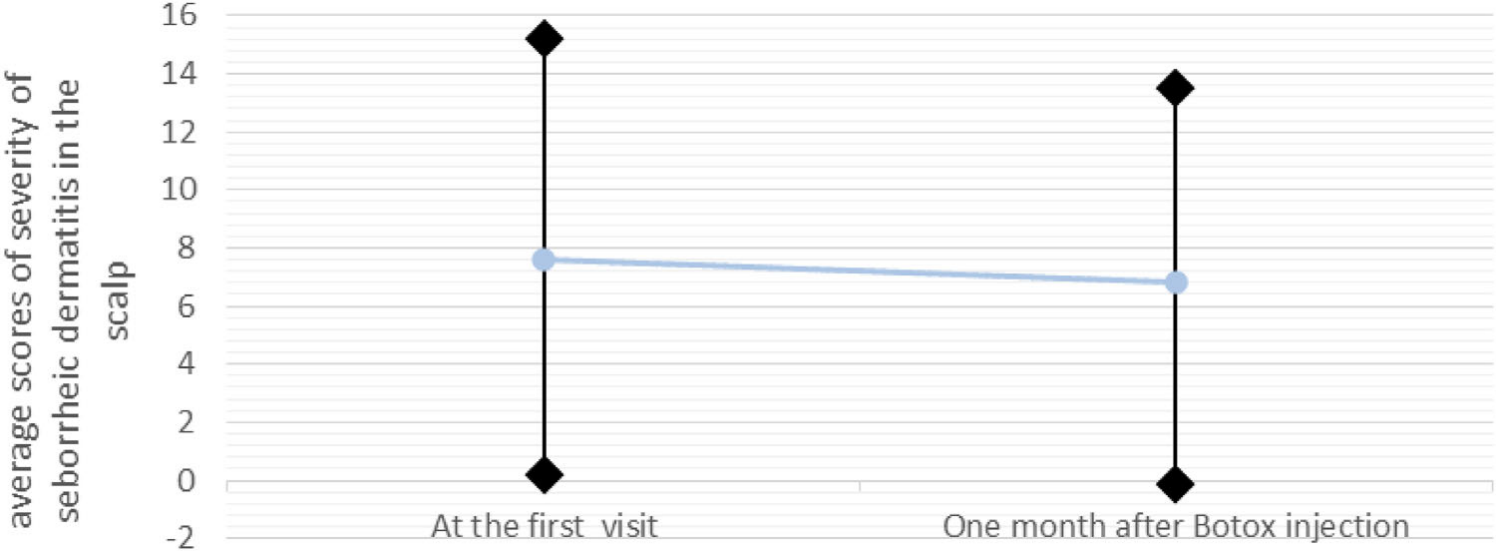
The chart of comparison between average scores of seborrheic dermatitis severity in the scalp at the first visit and one month after botulinum toxin injection.

The overall severity of seborrheic dermatitis in the scalp one month after Botox injection compared to the first visit, despite the average decrease from 7.7 (±7.15) to 6.6 (±6.76), there was no statistically significant difference (*p* value = 0.528) (Figure 3).

In the evaluation of the facial skin, despite the decrease in the average scores in all criteria of intensity and extent of lesions, in none of the criteria of skin erythema (*p* value = 0.317), skin sebum (*p* value = 0.317), scaling (*p* value = 0.083) and extent of lesions (*p* value = 0.317); there was no statistically significant difference.

**FIGURE 4:**
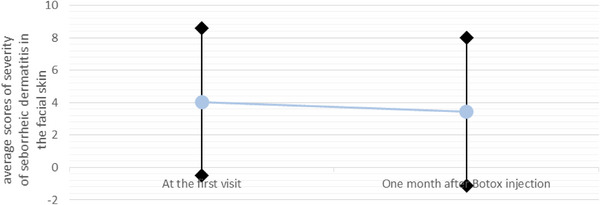
The chart of comparison of average scores of seborrheic dermatitis severity in the facial skin at the first visit and one month after botulinum toxin injection.

The overall severity of seborrheic dermatitis in the facial skin one month after botulinum toxin injection compared to the first visit, despite the decrease of the average from 4.05 (±4.55) to 3.45 (±4.55), there was no statistically significant difference (*p* value = 0.088) (Figure 4).

## 4 DISCUSSION

A common skin disorder, seborrheic dermatitis is a chronic recurrent inflammatory skin disease clinically manifested by scaly and erythematous patches with indistinct borders.^1^, ^2^, ^9^ Seborrheic dermatitis is a clinical diagnosis based on the location and appearance of the lesions.^3^ In most cases, the scalp, the middle part of the face and the upper part of the chest, which is rich in sebaceous glands, are affected, with symptoms such as dandruff, redness, itching, scaling and oiliness of the skin.^1^, ^4^

The pathophysiology proposed for the occurrence of seborrheic dermatitis states that a non‐immunological stimulation of the production of unsaturated fatty acid deposits on the skin surface is the cause of this disease.^9^

Most previous studies have evaluated the effect of botulinum toxin, emphasizing the reduction of sebum production by the sebaceous glands after the action of botulinum toxin. In addition, its role in reducing the symptoms of seborrheic dermatitis has been demonstrated by the intradermal injection of this substance.^10^, ^11^

On the other hand, in a 2013 study by Amy E Rose et al., with the aim of investigating the effect of intradermal botulinum toxin injection on the improvement of oily skin, 25 patients who were injected with botulinum toxin in the forehead area were studied. As a result of this study, it was found that intradermal injection of botulinum toxin in the forehead area was associated with a significant decrease in sebum production and 91% patient satisfaction.^12^

Also, in a study conducted in 2018 by Maria Shirshakova and colleagues on 12 patients with an average age of 35 years, with the aim of investigating the effect of using botulinum toxin type A for treatment of facial seborrhea, it was shown a positive and statistically significant relationship between the injection of this toxin and improvement of skin seborrhea.^13^

Previous researches have investigated the mechanism of action of this toxin; In a study conducted by Kyoung Rho‐Nark et al. in 2021 with the aim of investigating the effect of botulinum neurotoxin type A on the treatment of seborrhea of face, it was concluded that this toxin can stimulate the transmission of cholinergic messages in the autonomic nervous system and affect sebaceous glands reduce the production of sebum and thus be used in the treatment of diseases caused by excessive activity of sebaceous glands.^14^

In the results of the present study, while evaluating the information related to 20 patients referred to the skin clinic with an average age of 40.05 (±11.56) years who underwent botulinum toxin injection to remove facial wrinkles and were simultaneously suffering from to seborrheic dermatitis on the scalp or face, despite the reduction of the average scores in all the examined criteria of seborrheic dermatitis symptoms in the examination 1 month after botulinum toxin injection, no significant effect of using this toxin on the improvement of patients' symptoms was seen (*p* value >0.05). This result is not consistent with the results of the discussed studies. The quantity and nature of the investigated population, including the number of investigated patients, the average age and dominant gender of the patients, were not much different in our study and other discussed studies. However, investigating the causes of this difference in the results, the time of follow‐up and re‐visit of patients has been effective in evaluating the residual effect of Botox injection on symptoms.

While in the study of Amy E Rose et al. in 2013, the first follow‐up session of patients was done one week after Botox injection and was associated with a 75% reduction in sebum production^12^; In the present study, this time period was increased to one month after Botox injection, which is effective on our evaluation of the improvement of patients' symptoms.

Another influential factor is the injection technique and toxin access to the sebaceous glands; in this way, the shallowness of the injection depth may cause its ineffectiveness, and its deepness can lead to paralysis of the deep skeletal muscles.

It's worth noting that in Sapra et al.’s 2017 study, which compared the efficacy of intradermal and intramuscular botulinum toxin A injections for treating skin aging symptoms in ten women aged 35 to 65 years, no significant reduction in skin sebum secretion was observed.^15^ These findings align with our own study results. In this regard, the case report study written by Urbina reported an increase in sebum secretion and subsequent acne on one half of the face in a patient with unilateral facial paralysis.^16^

Based on this, it is possible to imagine a dual role for botulinum toxin in its effect on seborrheic dermatitis. The hypothesis that can be proposed is that intradermal injection of botulinum toxin, with the previously mentioned mechanisms, reduces the incidence and improves seborrhoeic dermatitis lesions. On the other hand, intramuscular injection of botulinum toxin causes muscle paralysis and delays the complete discharge of sebum, leading to the aggravation of seborrheic dermatitis symptoms. In our study and Sapra's study,^15^ while the symptoms of seborrheic dermatitis improved, the improvement was not significant. This could be due to the double effect of botulinum toxin on the symptoms of seborrheic dermatitis.

## 5 CONCLUSION

In our study, despite the intramuscular injection of Botox with cosmetic purposes for patients, the symptoms were associated with a slight reduction, which was not statistically significant. Therefore, in addition to correcting the follow‐up intervals, paying attention to the standardization of the injection technique and the type of toxin used can be one of the requirements for the design of future researches. Also, removing the factors confounding the results, such as examining the symptoms of the disease that overlap with other diseases and using a larger statistical population, will help to discover this relationship more clearly in the future.

## ETHICS STATEMENT

This study registered in the Ethics Committee and code of the study is ‘IR.IUMS.FMD.REC.1401, 372’. The researchers were committed and adhered to the principles of the Helsinki Convention and the Ethics Committee of the Iran University of Medical Sciences in all stages.

## Data Availability

All data produced in the present study are available upon reasonable request to the authors.
